# The construction of ventilation turrets in *Atta vollenweideri* leaf-cutting ants: Carbon dioxide levels in the nest tunnels, but not airflow or air humidity, influence turret structure

**DOI:** 10.1371/journal.pone.0188162

**Published:** 2017-11-16

**Authors:** Florian Halboth, Flavio Roces

**Affiliations:** Department of Behavioral Physiology and Sociobiology, Biocenter, University of Würzburg, Würzburg, Germany; The University of Auckland, NEW ZEALAND

## Abstract

Nest ventilation in the leaf-cutting ant *Atta vollenweideri* is driven via a wind-induced mechanism. On their nests, workers construct small turrets that are expected to facilitate nest ventilation. We hypothesized that the construction and structural features of the turrets would depend on the colony’s current demands for ventilation and thus might be influenced by the prevailing environmental conditions inside the nest. Therefore, we tested whether climate-related parameters, namely airflow, air humidity and CO_2_ levels in the outflowing nest air influenced turret construction in *Atta vollenweideri*. In the laboratory, we simulated a semi-natural nest arrangement with fungus chambers, a central ventilation tunnel providing outflow of air and an aboveground building arena for turret construction. In independent series, different climatic conditions inside the ventilation tunnel were experimentally generated, and after 24 hours, several features of the built turret were quantified, i.e., mass, height, number and surface area (aperture) of turret openings. Turret mass and height were similar in all experiments even when no airflow was provided in the ventilation tunnel. However, elevated CO_2_ levels led to the construction of a turret with several minor openings and a larger total aperture. This effect was statistically significant at higher CO_2_ levels of 5% and 10% but not at 1% CO_2_. The construction of a turret with several minor openings did not depend on the strong differences in CO_2_ levels between the outflowing and the outside air, since workers also built permeated turrets even when the CO_2_ levels inside and outside were both similarly high. We propose that the construction of turrets with several openings and larger opening surface area might facilitate the removal of CO_2_ from the underground nest structure and could therefore be involved in the control of nest climate in leaf-cutting ants.

## Introduction

The construction of underground nests provide animals with protection against predators and unfavorable climatic conditions, yet compromise the air exchanges between the nest environment and the atmosphere. To cope with such reduced air exchanges, two kinds of adaptive responses of the inhabitants are conceivable: first, a physiological adaptation, such as an increased tolerance to hypercapnic and hypoxic conditions, i.e., to high CO_2_ and to low O_2_ levels, respectively. And second, a behavioral adaptation, such as responses that help ventilating the nest via active or passive mechanisms, like fanning in bumblebees [1] or the construction of specific ventilatory nest structures [2].

Leaf-cutting ants of the genus *Atta* offer a particularly interesting model system for the study of such adaptations, since their nests are among the largest structures found in the animal kingdom. Depending on the species, leaf-cutting ant nests consist of a vast number of underground tunnels and chambers [3,4], in which the ants rear a symbiotic fungus as main food source for their brood. The fungus has strict demands of high humidity close to saturation and temperatures between 25°C and 30°C [5], and deviations from those ranges might be detrimental for fungal growth [6]. Colonies are able to maintain suitable microclimatic conditions inside the nest in two different ways, i.e., short-term behavioral responses and long-term modifications of the nest architecture.

Short-term behavioral reactions to unfavorable microclimatic conditions in leaf-cutting ants may include the collection of water in order to increase nest humidity [7] or the relocation of brood or fungus along temperature gradients in the nest [8], a behavior also found in other ant species [9]. In the long-term, workers might modify existing underground structures, i.e., enlarge tunnels or chambers, or excavate new ones when the current climatic conditions inside the nest are suboptimal. In *Acromyrmex lundii*, workers stop digging when soil temperature is lower than 20°C or rises above 30°C, and shift their digging activity to locations with more suitable temperatures [10]. This mechanism is likely to be involved in the determination of nest depth as leaf-cutting ants tend to avoid superficial soil layers and prefer to excavate their nests in deeper and cooler layers.

**Fig 1:**
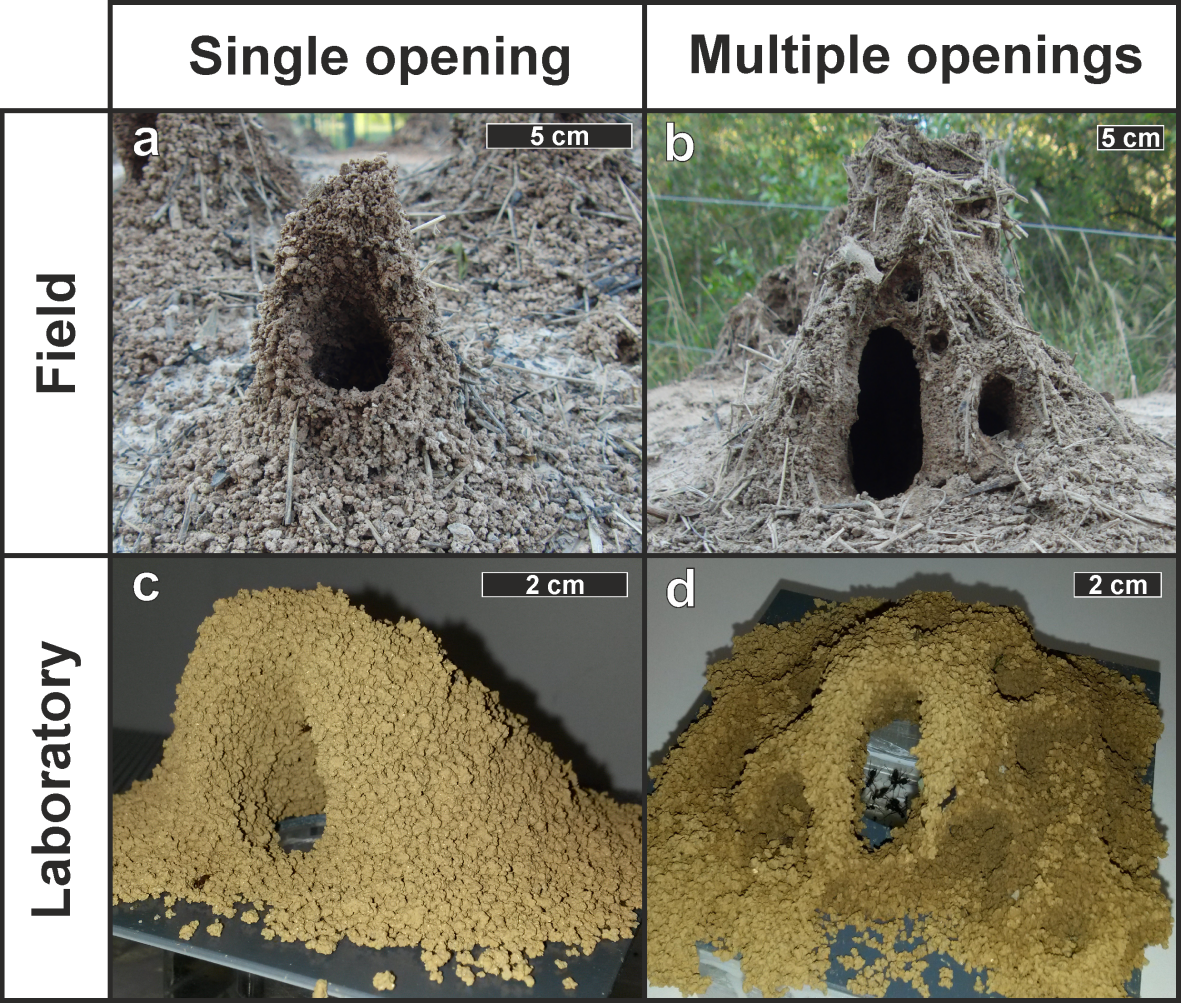
Ventilation turrets of *Atta vollenweideri*. Top row: Ventilation turrets of field nests (Formosa, Argentina) with a) one single opening and b) multiple openings. Bottom row: turrets constructed in a laboratory colony with c) one single opening and d) multiple openings. Note the different scale bars.

However, nesting in deeper soil layers might also entail disadvantages, as the carbon dioxide concentrations in the underground drastically increase with increasing depth [11]. Additionally, large amounts of carbon dioxide are produced underground due to the respiration of the colony and the decomposition of organic matter in the nest, and recent studies characterized the contributions of ant communities to the CO_2_ efflux from subterranean nests to the atmosphere, not only for leaf-cutting ants [12], but for other ant species as well [13]. Measurements in the field showed that, depending on soil properties like moisture and porosity, carbon dioxide concentrations in ant nests can reach values of 0.2% in *Pogonomyrmex badius* [14], about 1.5 to 4.5% in *Atta capiguara* or *Atta laevigata* [15] and up to 5.7% in *Atta vollenweideri* [16], and far exceed the atmospheric levels of currently 0.04% CO_2_. Especially for *Atta vollenweideri*, a species native to the clay-heavy soils of the Gran Chaco region in South America, the elevated carbon dioxide levels in the nest pose a major problem. Clay-heavy soils show low porosity and low air permeability [17], which hinders the removal of CO_2_ from underground chambers and the supply of the nest with oxygen via diffusion with the surrounding soil. To facilitate gas exchanges, nests of *Atta vollenweideri* rely on a wind-induced ventilation mechanism taking advantage of the Bernoulli principle [18], similar to that involved in the ventilation of prairie dogs’ burrows [19]. The nests possess an aboveground nest mound permeated with up to 200 nest openings that are not all used as exits or entrances by the ants [20,21]. Inflow and outflow of air through the nest openings depend on their location on the nest mound. Surface wind is dragging air out of central tunnels, followed by an inflow of air at the periphery [18]. On top of central nest openings, the ants construct conspicuous turrets that are expected to enhance nest ventilation by elevating the tunnel opening and exposing them to greater wind velocities (Fig 1A and 1B).

Most of the building material used for the construction of turrets originates from the excavation of nest structures in the underground. Workers carry soil pellets from underground digging sites to the surface and deposit them around the nest openings, resulting in the formation of crater-like soil heaps, a common feature of ant nests in warmer climates [22]. Additionally, workers rearrange the soil pellets and import building material like twigs and grasses from the immediate vicinity to form such particular structures [23,24]. The variables that lead to turret construction and influence the turret shape, however, are still largely unknown. Jonkman observed an increase in turret height on *Atta vollenweideri* nests after heavy precipitation, and also the closure of some turret openings during rain or during the winter months [20], indicating that turret construction is influenced by environmental conditions.

We hypothesized that the ants’ building behavior and the resulting structure of the turrets would depend on the colony’s current ventilation demands and thus might be influenced by the prevailing climatic conditions inside the nest. In the present study, we investigated turret-building behavior in the leaf-cutting ant *Atta vollenweideri* and the influence of climate-related parameters, i.e., airflow, air humidity and carbon dioxide levels in the outflowing nest air on the construction and structural features of the ventilation turrets.

## Material and methods

Experiments were carried out at the University of Würzburg, Germany from January to April 2015 and January to March 2016 using a laboratory colony of *Atta vollenweideri* founded in the year 2004 in Formosa, Argentina. The colony was kept under controlled conditions at 24.7 ± 0.5°C, 40 ± 5% air humidity and a 12h:12h LD cycle, and fed daily with fresh leaves of blackberry (*Rubus fruticosus*) and dog rose (*Rosa canina*). At the time of the experiments, the colony contained ca. 60–70 l fungus garden, distributed in 16 (at the end 19) fungus chambers made of plastic boxes. For data collection, individual fungus chambers were detached from the colony and connected to the experimental setup. Experiments were performed simultaneously on two setups containing four fungus chambers each. Since we used only one colony, both setups were located on opposite sides and connected to the same foraging arena. However, the two setups were treated as independent replicates, since we never observed the exchange of building material between them. In between experimental trials, the position of two of the four fungus chambers used in each setup was exchanged and in addition, one of the chambers was replaced by another fungus chamber from the main colony on a weekly basis to randomize sampling during data collection. Overall, we used 18 fungus chambers arranged in 16 different configurations. The two setups were connected with the main colony via PVC tubes during weekends, in order to allow the exchange of workers within the colony.

### General setup

The rationale of this study was to investigate turret construction and the influence of different environmental conditions inside the nest tunnels on the colony’s building response. We performed different experimental series focusing on some of the most important environmental variables prevailing in *Atta vollenweideri* leaf-cutting ant nests, i.e., the presence or absence of airflow in the nest tunnels, and both the relative humidity and the carbon dioxide concentration of the air leaving the nest.

**Fig 2:**
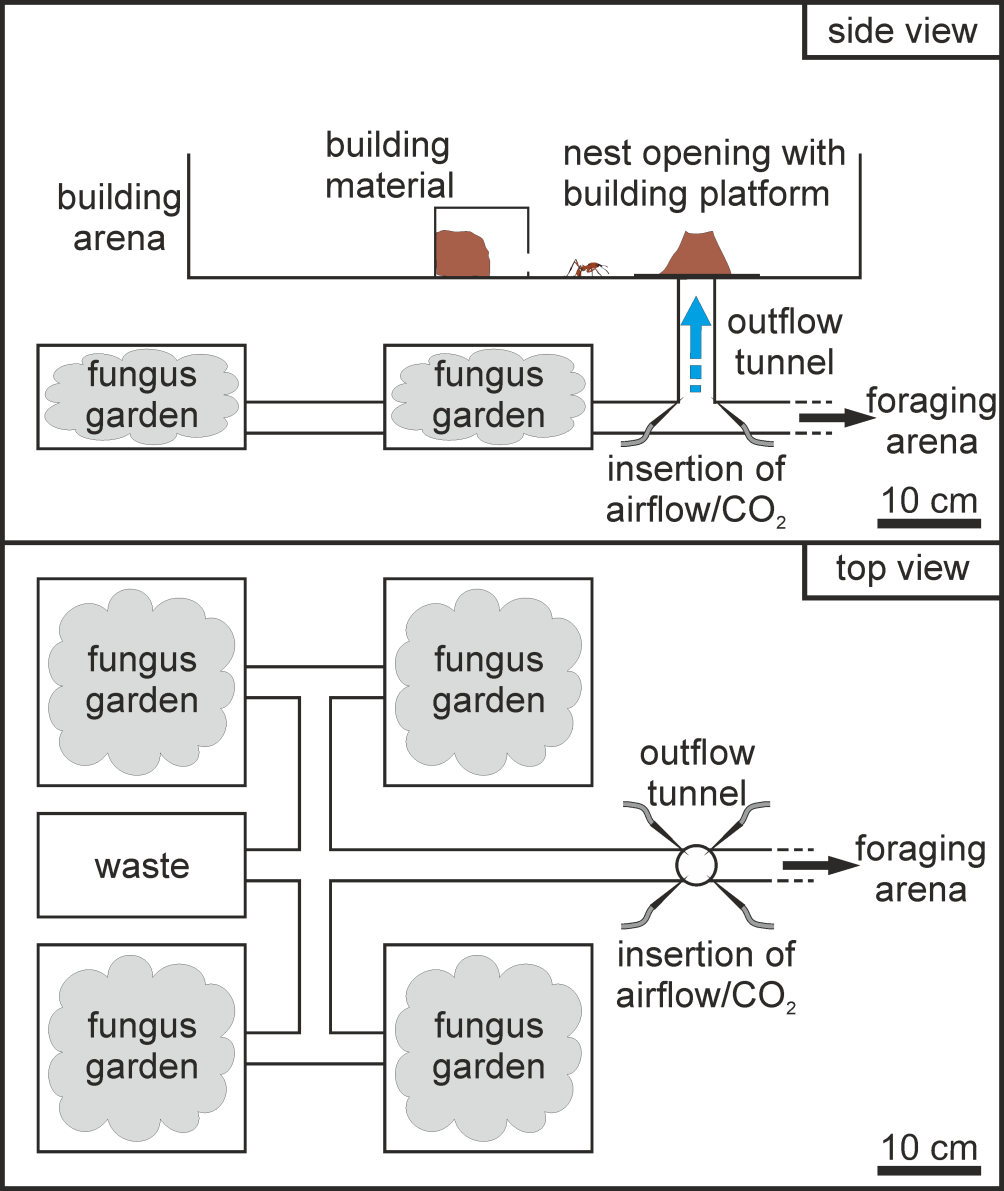
Experimental setup for the investigation of turret construction at an opening located above the nest. Side view: The nest was connected to a vertical tube leading to a building arena where a mixture of clay and sand was offered as building material. Inside the tube, an airflow with high or low humidity, and with varying levels of carbon dioxide depending on the experiments, was generated to investigate the effect of different environmental conditions in the outflowing nest air on the structure of the turret to be constructed on the building platform. Top view: Waste and fungus chambers simulating the natural arrangement of different nest compartments connected to an outflow tunnel, as observed in field nests. Note: The building arena is not depicted for the sake of clarity.

By performing experiments in the laboratory, we were able to manipulate one parameter at a time without changing the others. We used an experimental setup (Fig 2) that simulated the spatial arrangement of compartments observed in field nests, i.e., an underground area with fungus and waste chambers connected to the surface by tunnels that are either used by foragers as exits/entrances or serve ventilation purposes [21]. A similar setup has already proven suitable for the observation of turret construction in the studied species [24].

The experimental nest consisted of four fungus chambers (20 x 20 x 10 cm) and a waste chamber (20 x 10 x 10 cm) interconnected by transparent PVC tubes (Ø 3 cm) as depicted in Fig 2. A foraging arena was available at a distance of 120 cm, where the colony was provided daily with water and fresh leaves. Approximately 30 cm away from the nest a vertical plastic tube (Ø 3.3 cm, h = 15 cm) led to an open building arena (65 x 45 x 12 cm), simulating a vertical nest tunnel leading to the surface. The walls of the building arena were coated with Fluon^®^ to prevent the ants from escaping. Inside the arena 300 g of building material was offered to the ants, consisting of a 2:1 mixture of clay powder (CLAYTEC “Lehm gemahlen 10.001”, particle size: ≤ 0.5 mm) and sand (DORSILIT® 9 “Kristall II”, particle size: 0.6–1.2 mm) with a water content of 19% of the total mass. In order to prevent desiccation, the material was kept in a closed plastic box (10 x 10 x 9 cm) accessible to the ants only through a small opening in the front (Ø 2 cm). A building platform (12 x 12 x 0.1 cm) was placed on top of the nest opening to allow the removal of the constructed turret at the end of the trial without damaging its structure.

At the beginning of each trial, access to the building arena was given to the colony and all ants could freely move across the setup for the entire duration of the trial. After a short time, workers started to excavate small particles from the offered clay mixture and deposited the pellets around the tunnel opening, ultimately leading to the construction of a dome-shaped turret on the building platform. After 24 hours, the turret was removed and its height and the number of openings quantified. Opening aperture was estimated by measuring its diameter horizontally (d_h_) and vertically (d_v_), and by calculating opening surface using the formula for the area of an ellipse: $A=\pi \times \frac{dh}{2}\times \frac{dv}{2}$.

Total turret aperture was calculated as the sum of the aperture of all turret openings. Additionally, turret mass was measured to the nearest 0.1 g using precision scales (Mettler PM3000) after overnight drying in an oven.

### Experiment 1 (E1)–Turrets constructed on nest openings containing no airflow, humid air or dry air

In the first experiment, we tested whether the presence or absence of outflowing air in a nest tunnel, and its humidity, influence the construction and structure of ventilation turrets. In order to simulate an outflow tunnel, airflow inside the vertical tube was generated using compressed air from the laboratory line and adjusted to 2500 ml/min (0.15 m^3^/h) using a mass flow meter (Flow Bar 8—Sable Systems). The airflow was directed to the experimental setup via rubber tubes (Ø 3 mm) ending in pipette tips that were inserted into the bottom part of the vertical tunnel (Fig 2), thus creating an outflow of air from the nest with a velocity of ca. 5 cm/s, close to the values measured in outflow tunnels of field nests [18]. Depending on the series the airflow was humidified by leading it first into a glass wash bottle and then into the vertical tunnel. Air humidity inside the vertical tunnel was measured prior to the trials with a thermo-hygrometer (range: 10–99%), and the adjusted airflow velocity was checked with an anemometer (Testo 405-v1, range 0–5 m/s, resolution: 0.01 m/s). Three series were conducted that differed in the velocity and humidity of the airflow present in the vertical outflow tunnel. In the first series, no airflow was added to the nest tunnel, and air humidity in the nest was kept high (0 cm/s, 80% RH). In the second series, an outflow of humid air (5 cm/s, 80% RH) was generated inside the nest tunnel, in order to test the effect of the airflow itself as compared to the previous series. In the third series, we tested the effect of reduced air humidity by generating an outflow of dry air (5 cm/s, 40% RH) in the tunnel.

### Experiment 2 (E2)–Turrets constructed on nest openings with outflowing air containing different carbon dioxide levels

In the second experiment, we tested whether different carbon dioxide concentrations inside the nest tunnel, as may occur because of varying degrees of nest ventilation, affect the workers building behavior and the resulting turret structure. While lower carbon dioxide levels of 1–2% are common in shallow soil layers and in well-ventilated underground nests, higher levels around 6% usually indicate poor nest ventilation [16]. In order to simulate variable demands for nest ventilation, carbon dioxide was added to the outflowing air in different concentrations, depending on the experimental series, via the laboratory gas line. The concentration was regulated with a mass flow meter as in the first experiment. The mixture of air and CO_2_ was humidified with the help of glass wash bottles to reach approximately 80% relative humidity. Prior to the experiments, the concentration inside the outflow tunnel was measured using a hand-held carbon dioxide meter (Vaisala CARBOCAP^®^ GM70). Even though the airflow containing carbon dioxide was inserted in the nest at the bottom part of the vertical tube (Fig 2), no increase of carbon dioxide could be detected in adjacent tunnels or other compartments of the nest, i.e., CO_2_-enriched air could only be perceived by workers either inside the vertical tube or close to the tube opening. We conducted three different series, all providing outflow of humid air (5 cm/s, 80% RH) yet with different levels of carbon dioxide, i.e., 1% CO_2_, 5% CO_2_ and 10% CO_2_. One of the previous series from Experiment 1 was taken into account for comparisons, since it investigated the effect of atmospheric levels of carbon dioxide (ca. 0.04%) at the same conditions (airflow of 5 cm/s, 80% RH).

### Experiment 3 (E3)—Turrets constructed on nest openings with outflowing air containing similar carbon dioxide levels to those of the outside air

**Fig 3:**
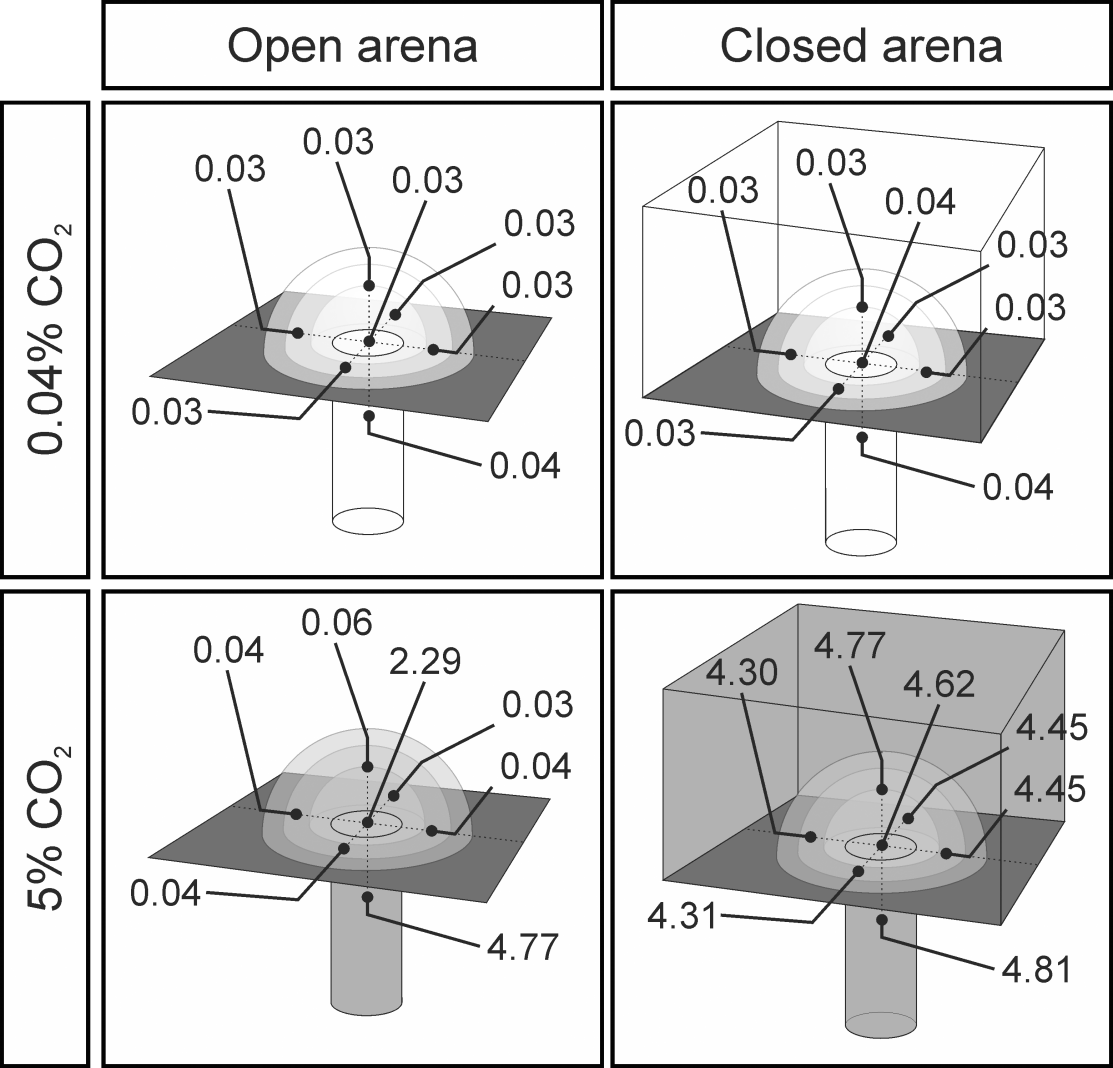
Schematic drawing of the experimental series in E3. Indicated is the carbon dioxide gradient at the building platform (rectangle) on top of the outflow nest tunnel (vertical tube). Black dots represent measuring points and the recorded mean CO_2_ levels (%), when gradients where either present (open arena, left column) or absent (closed arena, right column), both for the series using atmospheric (0.04%) or elevated CO_2_ levels (5%) in the outflowing air (top and bottom rows, respectively).

In the previous experiment E2, ants constructed turrets with multiple openings when the outflowing air contained high CO_2_ levels, as it will be presented in the Results. Therefore we investigated whether the construction of turrets with several minor openings depended on the strong differences in CO_2_ levels between the inside and the outside of the nest tunnel experienced by workers. In order to investigate whether a CO_2_ gradient is necessary for the construction of turrets with several openings, we performed four different series using the same setup as previously described. In the first two series, we provided outflowing air in the vertical nest tunnel with either atmospheric or 5% CO_2_-levels using an open building arena (Fig 3, left column), similar to the conditions of the previous experiment. In the third and fourth series (Fig 3, right column), we removed the CO_2_ gradient between the inside and the outside of the tunnel by covering the building arena with a Plexiglas plate (50 x 70 cm). This method allowed for a homogenous distribution of carbon dioxide in the whole arena. The third series provided outflow of atmospheric air in the closed arena (Fig 3, top right) and served therefore as a control for a potential effect of the cover on building responses. The fourth series provided outflow of 5% CO_2_ (Fig 3, bottom right) and no gradient between the inside and outside of the tunnel because of the cover. The carbon dioxide concentrations inside the building arena were measured for all four series prior to the experiments at different measuring points in a distance of ca. 3 cm around the tunnel opening (Fig 3). For the series using closed arenas, small holes, drilled in the cover of the building arena and closed during the actual trials, allowed the insertion of the hand-held carbon dioxide meter.

### Statistical analysis

All data was tested for normality using the Shapiro-Wilk normality test and consequently analyzed using either ANOVA followed by Tukey’s multiple comparison test (post-hoc) or Kruskal-Wallis test followed by Dunn’s multiple comparison test (post-hoc). Accordingly, either mean ± SD or median ± IQR values are presented for normally distributed or non-normally distributed data, respectively. However, for uniformity purposes, most results are graphically shown as boxplots representing median (horizontal bars), 25–75% percentiles (boxes) and min-max values (whiskers). The significance level was set to α = 0.05 for all tests.

## Results

Soon after allowing ants to enter the building arena they discovered the offered building material and initiated turret construction, i.e., workers started excavating small pellets from the clay/sand mixture that were subsequently transported, by themselves or other workers, to the building platform and placed around the nest opening. Initially, a ring of pellets was formed around the nest opening, which was later elevated to a conically-shaped structure containing a central opening, usually 2–3 cm in diameter. As the structure grew, however, the opening sometimes shifted laterally or several smaller openings emerged at the turret walls, depending on the series (Fig 1C and 1D). Experiments were finished after 24 hours, since the ants’ building activity gradually decreased and less workers were observed incorporating pellets into the turret. Preliminary observations of turret construction over 48 or 72 hours showed only slight increases in turret size as compared to that reached after 24 hours, and sporadic deposition of dead fungus and unsuitable plant material on the building arena.

### E1 –Turrets constructed on nest openings containing no airflow, humid air or dry air

**Fig 4:**
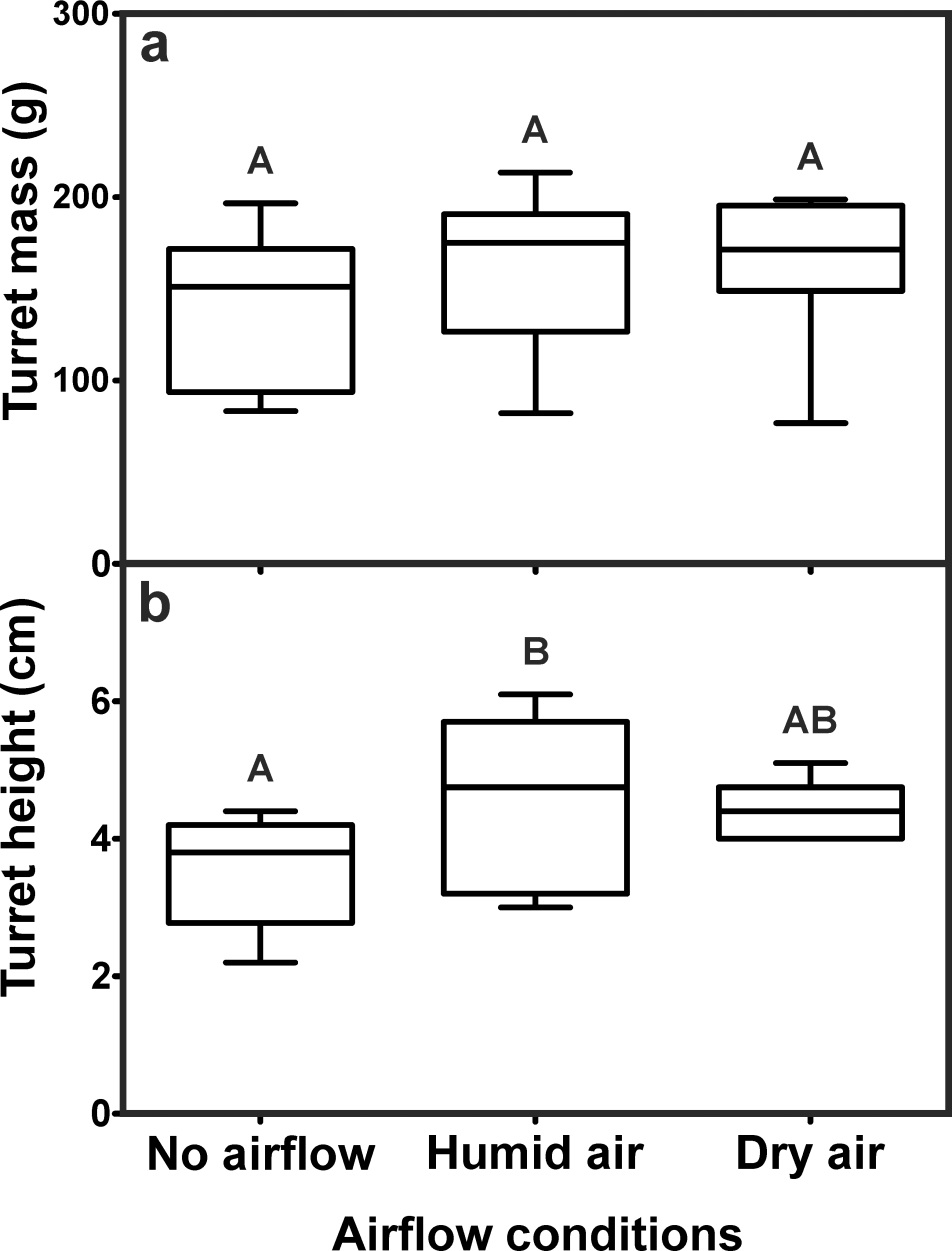
Influence of airflow and humidity on the construction of ventilation turrets. a) Turret mass (Kruskal-Wallis test: H_2,30_ = 1.99, p = 0.37). b) Turret height (ANOVA: F_2,27_ = 4.90, p = 0.015; Tukey’s Multiple Comparison test: No airflow vs. Humid air: p = 0.019, No airflow vs. Dry air: p = 0.055, Humid air vs. Dry air: p = 0.883). Each series comprises n = 10 replicates. Shown are medians (horizontal bars), 25–75% percentiles (boxes) and min-max values (whiskers). Groups sharing the same letters are not statistically different.

The overall building activity of the ants was roughly the same for all series performed, resulting in turrets of similar size and shape. Neither airflow, nor humidty affected the turret mass (Fig 4A). Turret dry mass was 151.2 ± 78.15 g (median ± IQR) when no airflow was generated inside the vertical tunnel, 175.3 ± 64 g (median ± IQR) when the air was humidified and 171.6 ± 46.5 g (median ± IQR) when dry air was presented in the outflow tunnel (for statistics see Figure caption).

The height of the constructed turrets, however, showed significant differences among the series (Fig 4B). Without airflow the average turret height was 3.50 ± 0.76 cm (mean ± SD), but significnatly increased to 4.60 ± 1.18 cm (mean ± SD) when outflow of humid air was present at the nest opening. Outflow of dry air at the nest opening resulted in the construction of intermediate sized turrets averaging 4.42 ± 0.41 cm (mean ± SD), a value not statistically different from those of the two other series.

**Fig 5:**
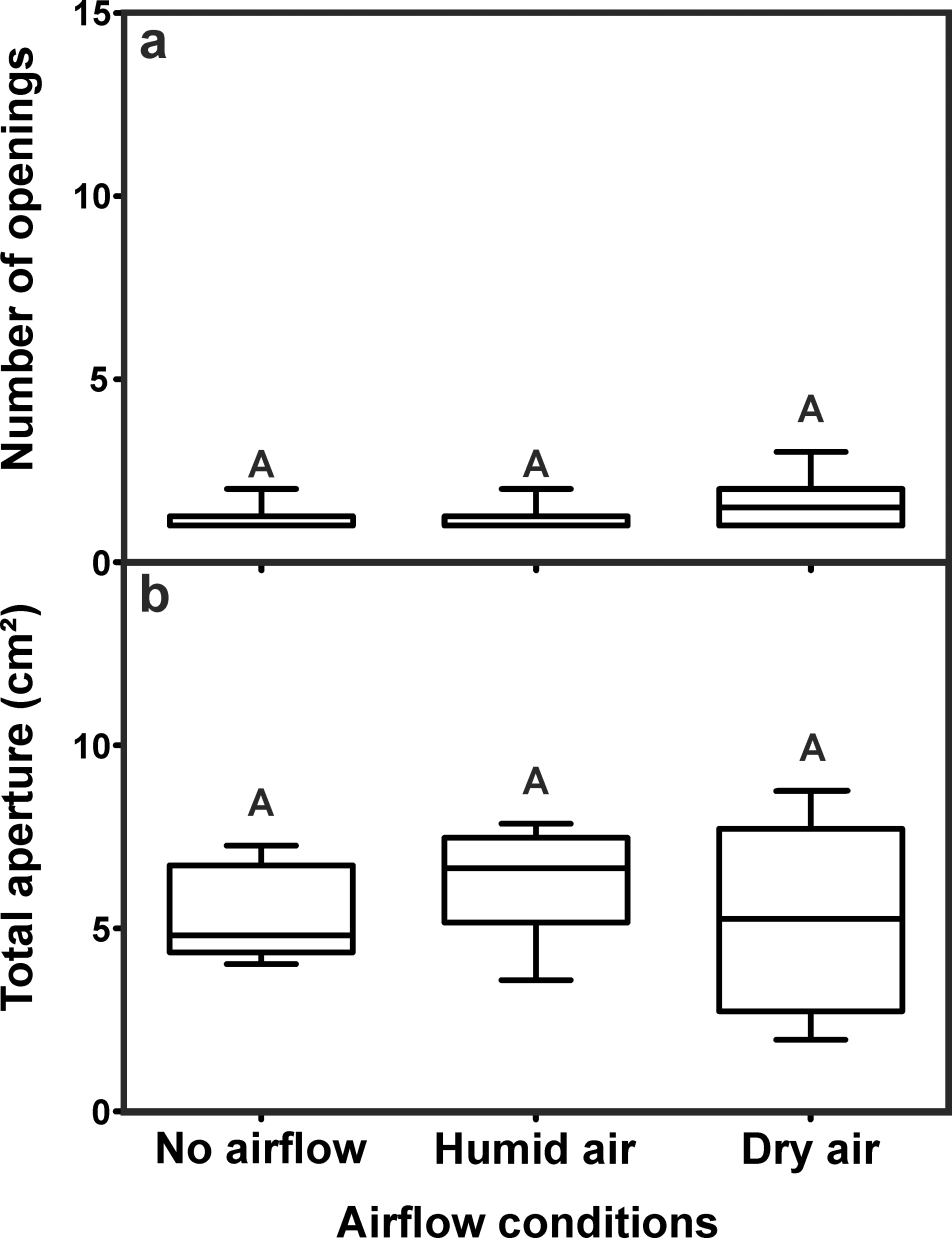
Influence of airflow and humidity on the structure of the ventilation turret. a) Number of turret openings (Kruskal-Wallis test: H_2,30_ = 3.10, p = 0.21). b) Total turret aperture (ANOVA: F_2,27_ = 0.81, p = 0.46). Each series comprises n = 10 replicates. Shown are medians (horizontal bars), 25–75% percentiles (boxes) and min-max values (whiskers). Groups sharing the same letters are not statistically different.

Concerning the structural features of the turrets, no statistical differences were found among the series, neither for the number of turret openings (Fig 5A), nor the total turret aperture (Fig 5B). Turrets had 1.0 ± 0.25 (median ± IQR) openings in the no airflow series and 1.0 ± 0.25 (median ± IQR), and 1.5 ± 1 (median ± IQR) openings in the humid air and dry air series, respectively.

Total turret aperture, i.e., the sum of the aperture of all openings, was also similar in all series with 5.37 ± 1.26 cm^2^ (mean ± SD) in the series without airflow, 6.23 ± 1.41 cm^2^ (mean ± SD) in the series with humid air, and 5.23 ± 2.69 cm^2^ (mean ± SD) in the series with dry air.

### E2 –Turrets constructed on nest openings containing different carbon dioxide levels

**Fig 6:**
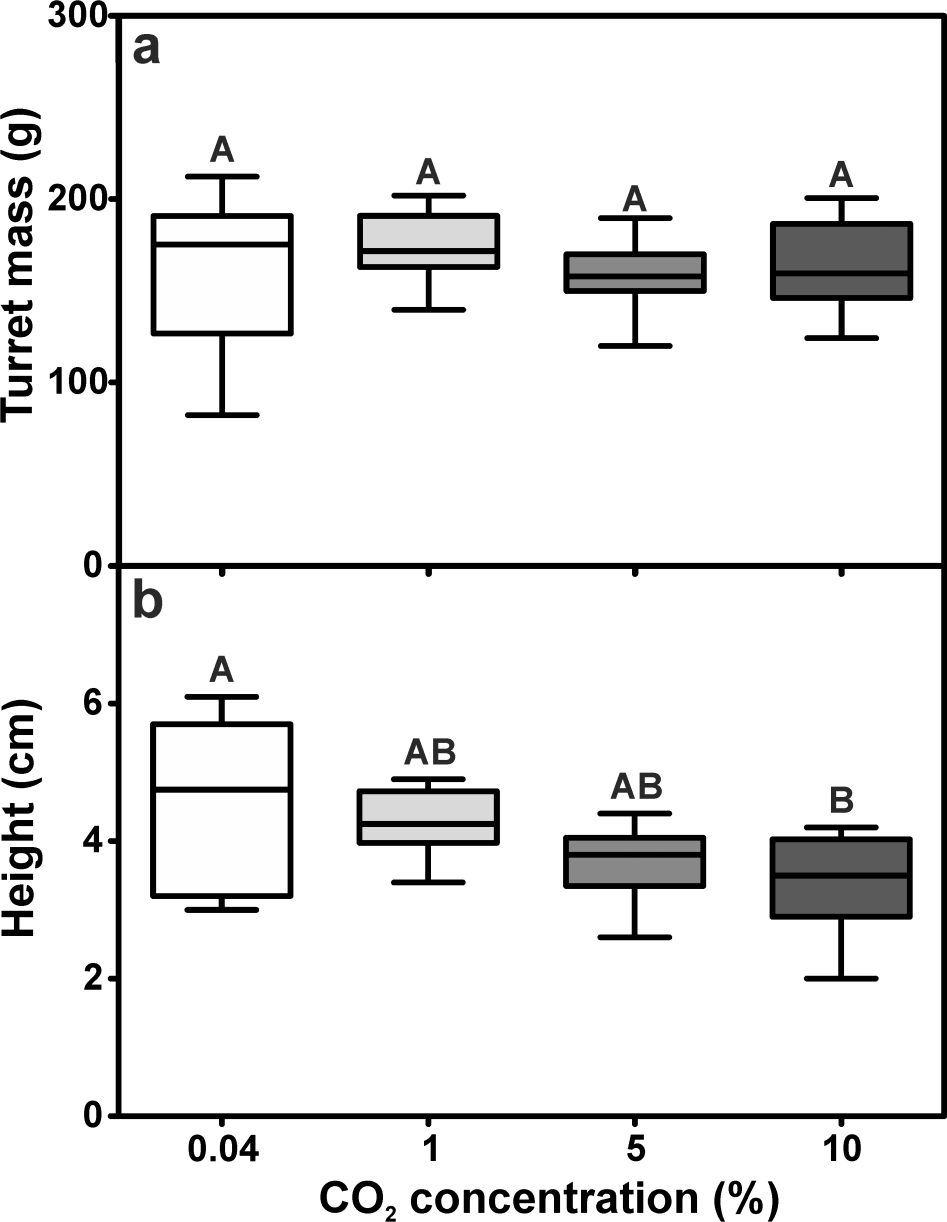
Influence of carbon dioxide levels on the construction of ventilation turrets. a) Turret mass (ANOVA: F_3,36_ = 0.46, p = 0.71). b) Turret height (ANOVA: F_3,36_ = 5.19, p = 0.004; Tukey’s Multiple Comparison Test: atmospheric vs. 1% CO_2_: p = 0.807, atmospheric vs. 5% CO_2_: p = 0.054, atmospheric vs. 10% CO_2_: p = 0.006, 1% CO_2_ vs. 5% CO_2_: p = 0.307, 1% CO_2_: vs. 10% CO_2_: p = 0.057, 5% CO_2_ vs. 10% CO_2_: p = 0.821). Each series comprises n = 10 replicates. Shown are medians (horizontal bars), 25–75% percentiles (boxes) and min-max values (whiskers). Groups sharing the same letters are not statistically different.

In the second experiment we simulated conditions of well-ventilated nests by adding 1% carbon dioxide, or of poorly-ventilated nests by adding either 5% or 10% carbon dioxide to the outflow in the vertical tunnel. The overall building activity of the ants was similar for all series, resulting in turrets of similar size and shape. As in the previous experiment, there were no differences in the amount of building material used for turret construction among the series (Fig 6A). Mean turret mass was 162.34 ± 41.22 g (mean ± SD) for the series using humid air without adding carbon dioxide (atmospheric CO_2_), 172.19 ± 18.94 g (mean ± SD) for 1% CO_2_, 158.33 ± 18.39 g (mean ± SD) for 5% CO_2_ and 163.28 ± 23.55 g (mean ± SD) for 10% CO_2_.

Turret height, however, was affected by the carbon dioxide concentration in the outflow tunnel (Fig 6B). Turrets of the series with atmospheric levels were the tallest, and turret height decreased with increasing carbon dioxide concentrations at the nest opening, resulting in the smallest turrets in the 10% CO_2_ series. The values averaged 4.60 ± 1.18 cm (mean ± SD) for atmospheric levels, 4.29 ± 0.47 cm (mean ± SD) for 1% CO_2_, 3.68 ± 0.56 cm (mean ± SD) for 5% CO_2_ and 3.38 ± 0.22 cm (mean ± SD) for the 10% CO_2_ series.

**Fig 7:**
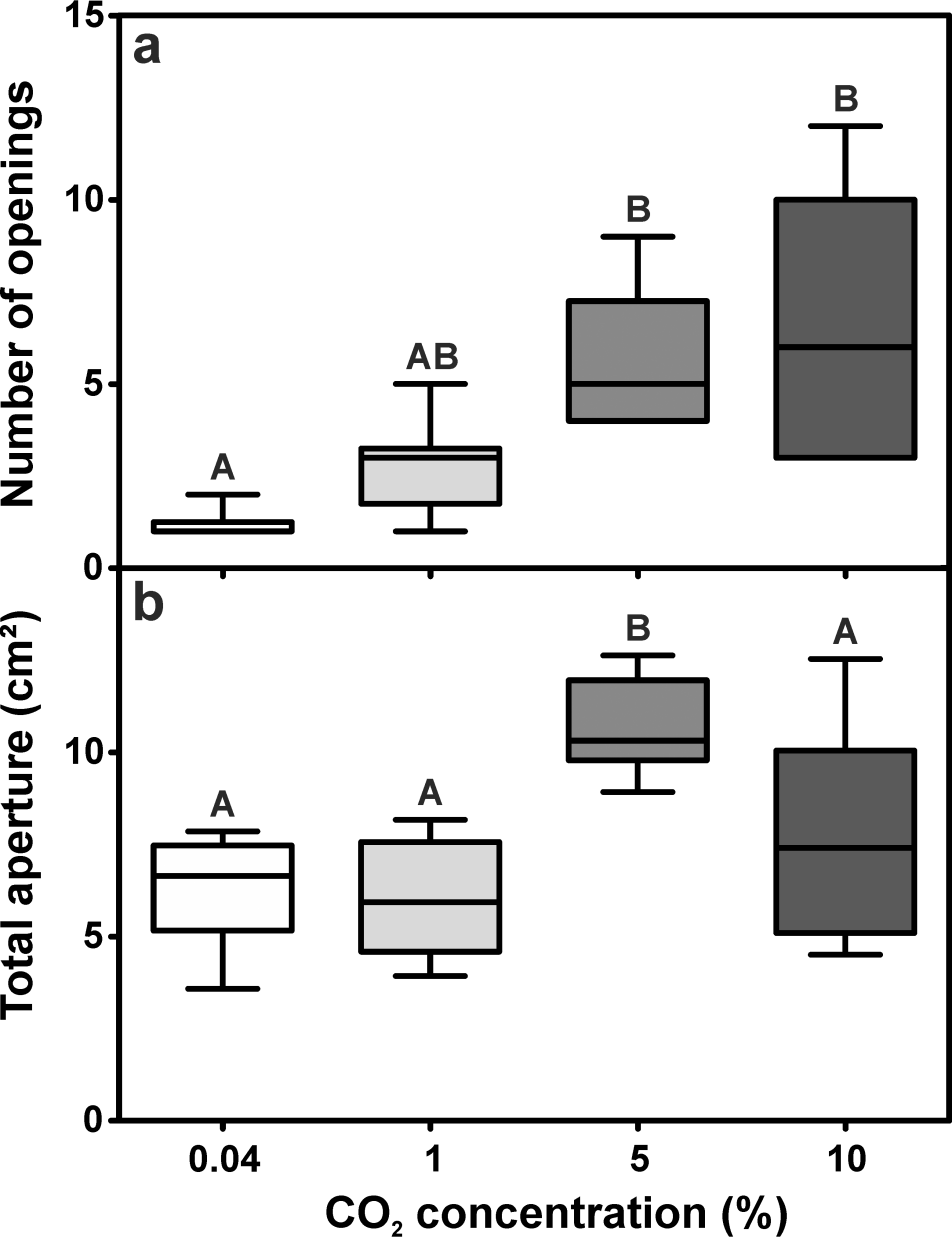
Influence of carbon dioxide levels on the structure of the ventilation turret. a) Number of turret openings (Kruskal-Wallis test: H_3,40_ = 27.81, p < 0.001; Dunn’s Multiple Comparison Test: atmospheric vs. 1% CO_2_: p = 0.501, atmospheric vs. 5% CO_2_: p < 0.001, atmospheric vs. 10% CO_2_: p < 0.001, 1% CO_2_ vs. 5% CO_2_: p = 0.057, 1% CO_2_ vs. 10% CO_2_: p = 0.053, 5% CO_2_ vs. 10% CO_2_: p = 1.000). b) Total turret aperture (ANOVA: F_3,36_ = 13.95, p < 0.001; Tukey’s Multiple Comparison Test: atmospheric vs. 1% CO_2_: p = 0.996, atmospheric vs. 5% CO_2_: p < 0.001, atmospheric vs. 10% CO_2_: p = 0.239, 1% CO_2_ vs. 5% CO_2_: p < 0.001, 1% CO_2_ vs. 10% CO_2_: p = 0.161, 5% CO_2_ vs. 10% CO_2_: p = 0.006). Each series comprises n = 10 replicates. Shown are medians (horizontal bars), 25–75% percentiles (boxes) and min-max values (whiskers). Groups sharing the same letters are not statsitically different.

The structural features of the ventilation turrets were strongly influenced by the carbon dioxide concentration at the nest opening, as the number of turret openings differed among the series (Fig 7A). While most turrets constructed under atmospheric CO_2_ levels had just one opening, turrets constructed under elevated carbon dioxide levels usually possessed one larger and several smaller openings. Turrets had 1.00 ± 0.25 (median ± IQR) openings for the series using atmospheric levels and 3.00 ± 1.50 (median ± IQR) openings for 1% CO_2_, but the number significantly increased to 5.00 ± 3.25 and 6.00 ± 7.00 (median ± IQR) openings for 5% CO_2_ and 10% CO_2_, respectively.

Alongside with the number of turret openings, the total aperture of the ventilation turrets was also affected by the different levels of carbon dioxide in the outflowing air (Fig 7B). Turret aperture was similar for low carbon dioxide concentrations and was 6.23 ± 1.41 cm^2^ (mean ± SD) in the series with atmospheric levels and 6.06 ± 1.58 cm^2^ (mean ± SD) for 1% CO_2_. However, it was significantly larger in the 5% CO_2_ series with 10.61 ± 1.20 cm^2^ (mean ± SD), but not in the 10% CO_2_ series with 7.76 ± 2.61 cm^2^ (mean ± SD).

**Fig 8:**
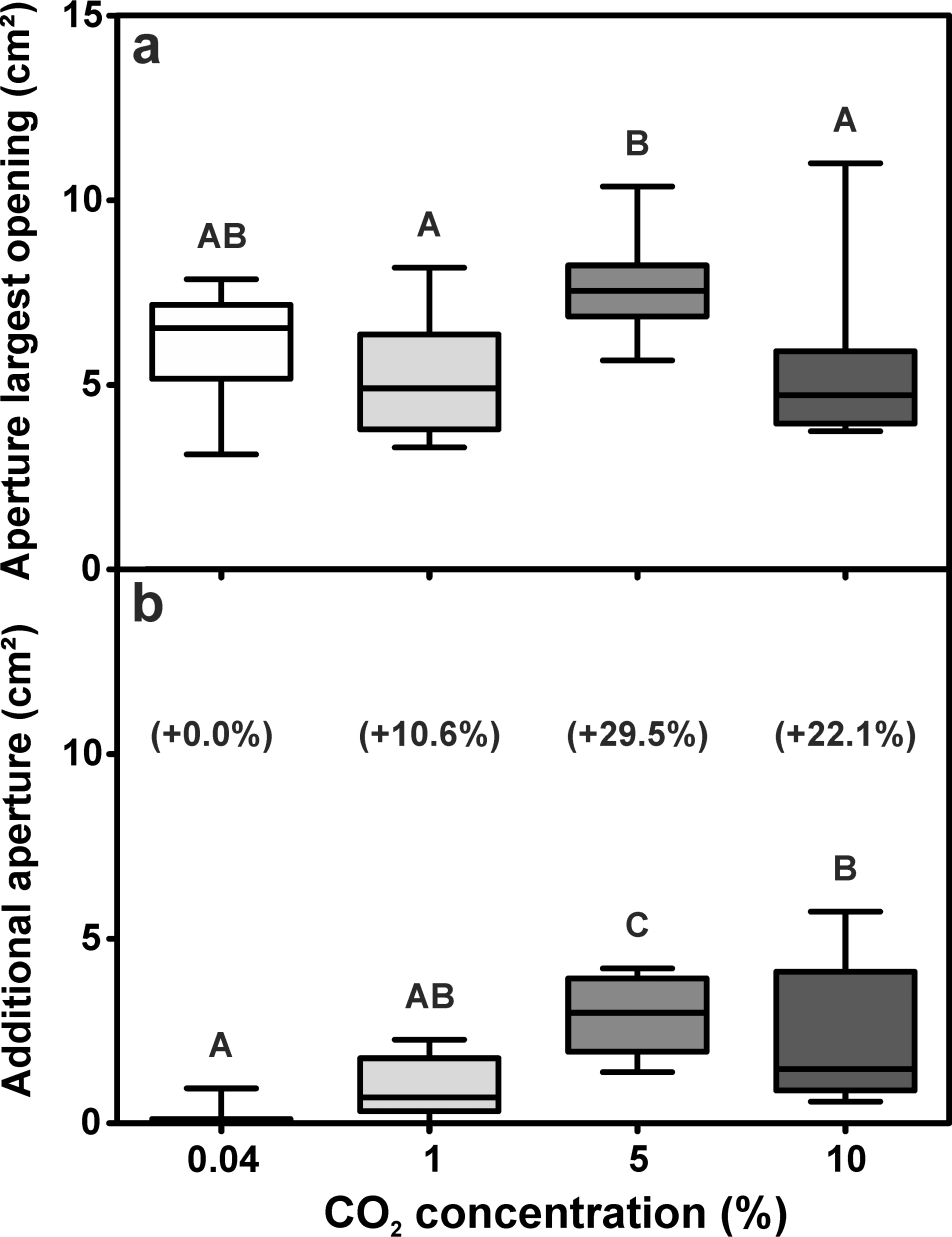
Influence of carbon dioxide levels on total turret aperture. a) Aperture of the single main opening (Kruskal-Wallis test: H_3,40_ = 13.43, p = 0.004; Dunn’s Multiple Comparison Test: atmospheric vs. 1% CO_2_: p = 1.000, atmospheric vs. 5% CO_2_: p = 0.250, atmospheric vs. 10% CO_2_: p = 1.000, 1% CO_2_ vs. 5% CO_2_: p = 0.008, 1% CO_2_ vs. 10% CO_2_: p = 1.000, 5% CO_2_ vs 10% CO_2_: p = 0.010). b) Sum of the apertures of all minor openings (Kruskal-Wallis test: H_3,40_ = 24.60, p < 0.001; Dunn’s Multiple Comparison Test: atmospheric vs. 1% CO_2_: p = 0.471, atmospheric vs. 5% CO_2_: p < 0.001, atmospheric vs. 10% CO_2_: p = 0.002, 1% CO_2_ vs. 5% CO_2_: p = 0.032, 1% CO_2_ vs 10% CO_2_: p = 0.424, 5% CO_2_ vs. 10% CO_2_: p = 1.000). Values in parentheses indicate the median increase in total turret aperture achieved by the addition of smaller openings. Each series comprises n = 10 replicates. Shown are medians (horizontal bars), 25–75% percentiles (boxes) and min-max values (whiskers). Groups sharing the same letters are not statistically different.

Total turret aperture comprised the aperture of the turrets’ largest opening and the aperture of the several smaller openings. Increasing carbon dioxide levels significantly influenced the aperture of the largest opening (Fig 8A). Comparable to the total turret aperture, the largest opening size was observed in the group containing 5% CO_2_ (7.54 ± 1.38 cm^2^; median ± IQR), while the main opening was smaller for the series with 1% and 10%, with 4.90 ± 2.57 cm^2^ (median ± IQR) and 4.71 ± 1.96 cm^2^ for 10% CO_2_, respectively, and intermediate for the series with atmospheric levels, with 6.54 ± 2.01 cm^2^ (median ± IQR).

Besides the largest opening, most turrets possessed several smaller openings that largely contributed to the total aperture of the ventilation turrets (Fig 8B). When the outflowing air contained atmospheric CO_2_ levels, turrets hardly had more than one large opening, resulting in the lowest average total aperture. However, the construction of several minor openings led to an increase of total turret aperture in the other series. The increase was especially prominent in the 5% CO_2_ and 10% CO_2_ series, where the carbon dioxide levels were elevated beyond the levels occuring in well-ventilated field nests. By the addition of several minor openings, turret aperture increased by 0.00 ± 0.12 cm^2^ (median ± IQR) for atmospheric levels, 0.70 ± 1.42 cm^2^ (median ± IQR) in the 1% CO_2_ series, 2.99 ± 1.99 cm^2^ (median ± IQR) in the 5% CO_2_ series and 1.47 ± 3.22 cm^2^ (median ± IQR) in the 10% CO_2_ series. The addition of multiple smaller openings therefore accounted for an increase in total turret aperture of up to ~30% as compared to the conditions when CO_2_ levels in the outflowing air were atmospheric (Fig 8B).

**Fig 9:**
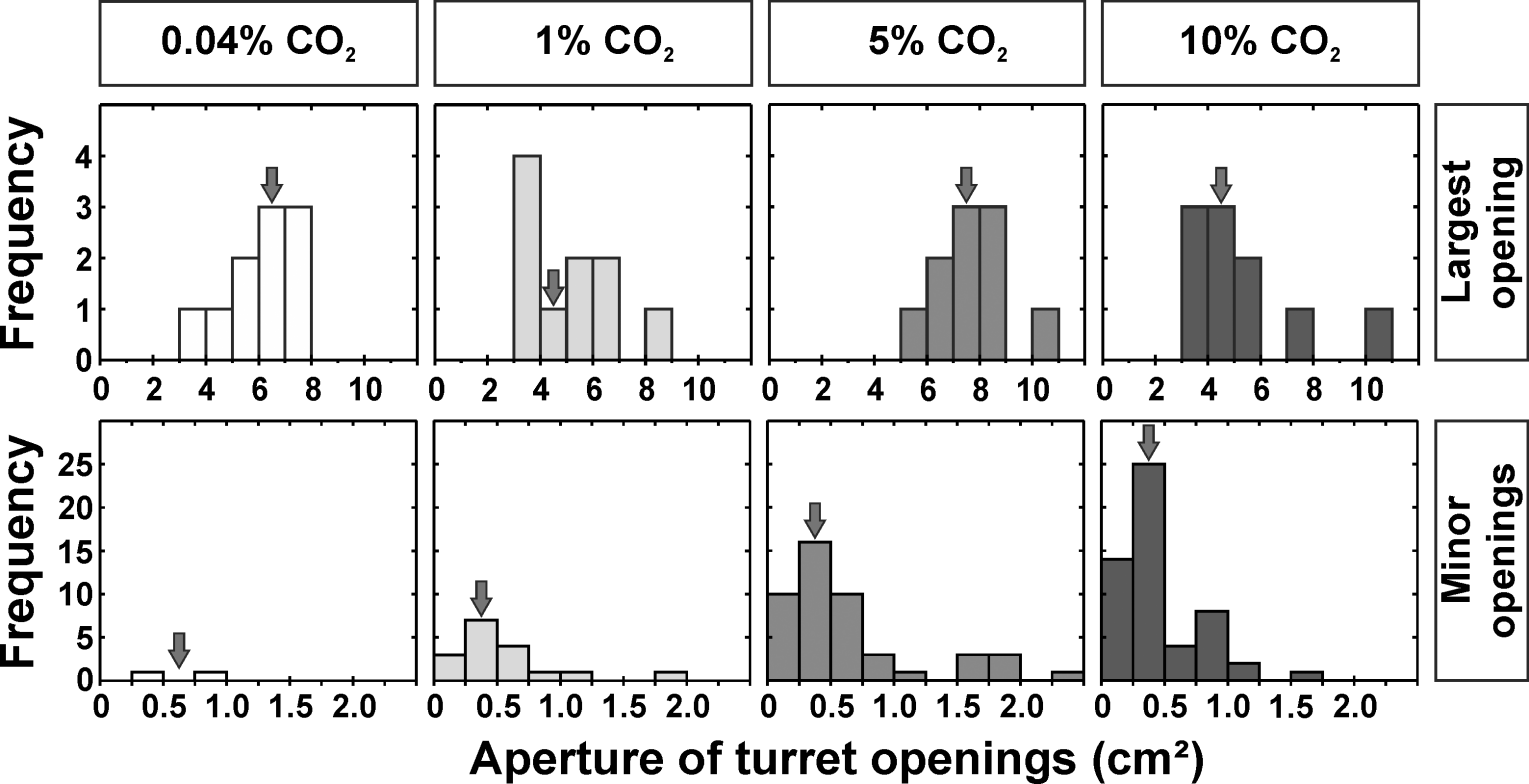
Size distribution of the largest opening (first row) and minor openings (second row) measured on turrets constructed on openings with outflowing air of different carbon dioxide levels. Arrows indicate median turret aperture, calculated for the largest openings (n = 10 each) and minor openings of the series using atmospheric CO_2_ levels (n = 2), 1% CO_2_ (n = 17), 5% CO_2_ (n = 47) and 10% CO_2_ (n = 54). Due to the differences in sample sizes, no statistical analysis was performed. Note the different scale on both x- and y-axis between the largest and the minor openings.

The size of the single largest turret opening strongly varied among trials in each series, yet it was markedly larger than the size of minor openings (Fig 9, top and bottom row, respectively). The median size of minor openings was 0.71 cm^2^ for atmospheric levels, 0.44 cm^2^ for 1% CO_2_, 0.38 cm^2^ for 5% CO_2_ and 0.38 cm^2^ for 10% CO_2_.

### Experiment 3 (E3)—Turrets constructed on nest openings with outflowing air containing similar carbon dioxide levels to those of the outside air

**Fig 10:**
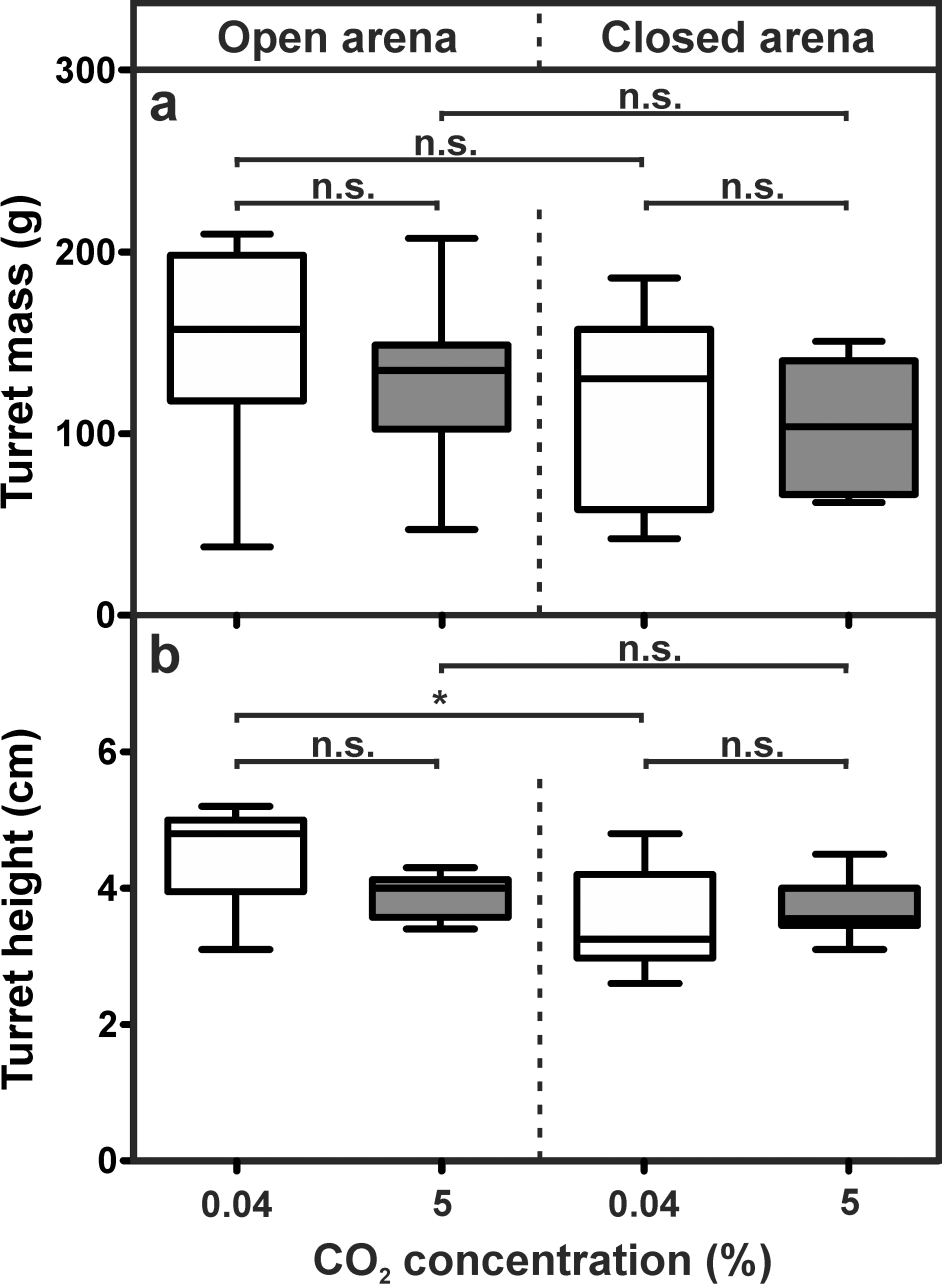
Influence of a carbon dioxide gradient on turret construction. a) Turret mass (Kruskal-Wallis test: H_3,40_ = 5.04, p = 0.169). b) Turret height (ANOVA: F_3,36_ = 5.55, p < 0.003; Dunn’s Multiple Comparison Test (post-hoc): open arena atmospheric CO_2_ vs. 5% CO_2_: p = 0.148, closed arena atmospheric CO_2_ vs. 5% CO_2_: p = 1.000, atmospheric CO_2_ open vs. closed: p = 0.002, 5% CO_2_ open vs. closed: p = 1.000). Each series comprises n = 10 replicates. Shown are medians (horizontal bars), 25–75% percentiles (boxes) and min-max values (whiskers). Asterisks indicate statistically significant differences between groups.

In the third experiment we investigated whether the construction of a turret with several minor openings depended on the strong differences in CO_2_ levels between the outflowing and the outside air. Covering the building arena to remove the CO_2_ gradient did not affect the overall building activity of the colony, as similar-sized dome-shaped turrets were constructed in all series. The mass of the turrets was similar in all four series (Fig 10A). When the building arena was open, turret mass averaged 157.30 ± 80.20 g (median ± IQR) for atmospheric CO_2_ levels and 134.80 ± 46.30 g (median ± IQR) for 5% CO_2_. Turret mass in closed arenas averaged 130.30 ± 99.07 g (median ± IQR) and 103.70 ± 73.70 g (median ± IQR) for atmospheric levels and 5% CO_2_, respectively.

Turret height was also independent of the presence of a CO_2_ gradient under most conditions (Fig 10B). There was only a significant difference in height between turrets constructed under atmospheric CO_2_ levels with a CO_2_ gradient (open arena: 4.46 ± 0.69 cm; mean ± SD) and without a gradient (closed arena: 3.50 ± 0.71 cm; mean ± SD). When carbon dioxide levels were elevated to 5%, turret height was 3.92 ± 0.31 cm (mean ± SD) for open and 3.69 ± 0.41 cm for closed arenas.

**Fig 11:**
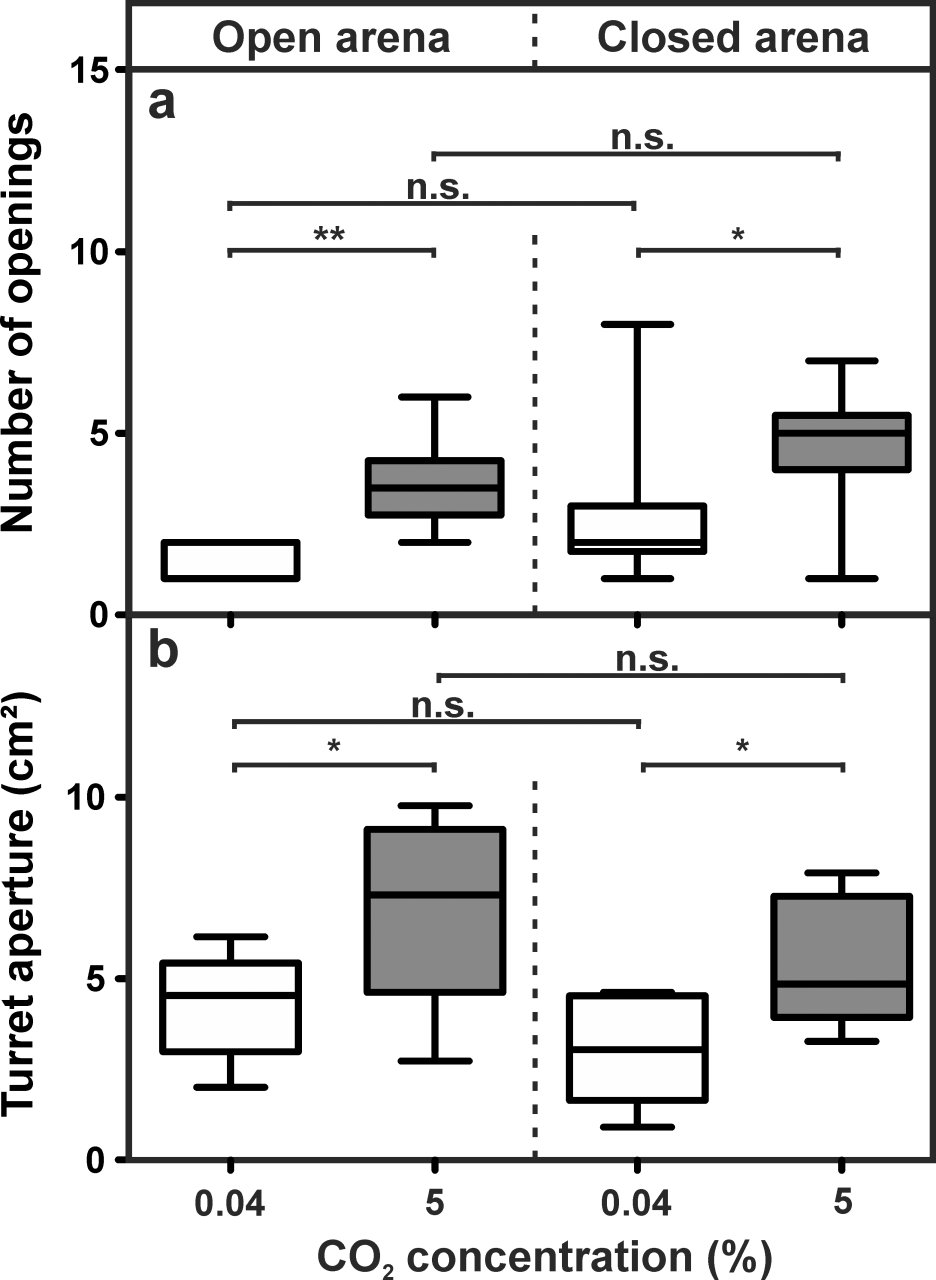
Influence of a carbon dioxide gradient on turret structure. a) Number of turret openings (Kruskal-Wallis test: H_3,40_ = 21.13, p < 0.001; Dunn’s Multiple Comparison Test (post-hoc): open arena atmospheric CO_2_ vs. 5% CO_2_: p = 0.003, closed arena 0% CO_2_ vs. 5% CO_2_: p = 0.046, 0% CO_2_ open vs. closed: p = 0.324, 5% CO_2_: open vs. closed: p = 1.000). b) Total turret aperture (ANOVA: F_3,36_ = 8.14, p < 0.001; Dunn’s Multiple Comparison Test (post-hoc): open arena 0% CO_2_ vs. 5% CO_2_: p = 0.011, closed arena 0% CO_2_ vs. 5% CO_2_: p = 0.021, 0% CO_2_ open vs. closed: p = 0.583, 5% CO_2_ open vs. closed: p = 0.369). Each series comprises n = 10 replicates. Shown are medians (horizontal bars), 25–75% percentiles (boxes) and min-max values (whiskers). Asterisks indicate statistical significant differences between groups.

Similar to the experiment E2, the structural features of the turrets significantly varied among the series (Fig 11A). The absence of a gradient, however, did not affect the number of turret openings, as there were no differences between open and closed arenas. Differences were only observed between series with atmospheric levels and the 5% CO_2_ series, as expected: in open arenas with a CO_2_ gradient, the number of turret openings was 1.00 ± 1.00 (median ± IQR) for atmospheric levels and 3.50 ± 1.50 (median ± IQR) for 5% CO_2_. When the gradient was removed, turrets had 2.00 ± 1.25 (median ± IQR) openings in the series using atmospheric levels and 5.00 ± 1.50 (median ± IQR) openings in the 5% CO_2_ series.

The increase in the number of turret openings was also accompanied by an increase in turret aperture (Fig 11B). Again, turret aperture was only larger when carbon dioxide concentrations were elevated. Covering the building arena, i.e., removing the gradient at the tunnel opening, did not affect turret structure and no difference was observed between series with open and covered arenas when CO_2_ levels were equal. Total turret aperture in open arenas was 4.28 ± 1.38 cm^2^ (mean ± SD) for atmospheric CO_2_ and 6.91 ± 2.50 cm^2^ (mean ± SD) for 5% CO_2_. Turrets constructed in covered arenas had a total aperture of 3.06 ± 1.43 cm^2^ (mean ± SD) in the series with atmospheric values and 5.50 ± 1.78 cm^2^ (mean ± SD) in the 5% CO_2_ series.

## Discussion

### Building behavior and turret construction

In all experiments, workers excavated and formed pellets from the offered building material and transported them to the building platform, where they constructed a dome-shaped turret around the nest opening. Although single pellets were occasionally dropped by workers in other places, construction behavior was never observed at locations in the building arena other than around the nest opening. Usually, a worker did not just drop the transported pellet at the nest opening, but rather searched for a suitable spot at the construction site and incorporated its pellet into the existing structure by pressing it onto the turret wall using their mandibles and front legs. A similar behavior is shown by leaf-cutting ant workers when soil pellets are deposited at the walls of underground tunnels or chambers during nest excavation [25] or during the incorporation of plant material into the fungus garden [26]. After the deposition of the pellet, workers often returned to the digging site in order to excavate or pick up another pellet and repeat the process. Although turret construction was quite slow at the beginning, more and more ants participated in excavation and transport and after a couple of hours, the turret grew in both height and diameter. Turret height and turret mass after 24 hours were considered as a reliable indicator for the colony’s overall building activity, as turrets usually had a similar size and shape across the experiments. However, the structural features of the turrets were much more variable among the series, depending on the prevailing climatic conditions at the nest opening. The carbon dioxide concentration and not the presence of airflow or the air humidity strongly affected the construction of the turrets and led to differences in both the number of turret openings and the total turret aperture among the series.

Noteworthy, workers always constructed turret-shaped structures on top of the nest opening in our experiments, independent of the climatic conditions inside the nest tunnel. This indicates that workers do not require outflow of air, a humidity gradient or carbon dioxide gradient at the nest opening in order to initiate turret construction. Although speculative, other factors might be used as cues for the initiation of turret construction by workers, as well. For example, volatiles, like colony odors or pheromones might leave through nest openings even without outflowing air and could be used by workers on the mound as orientation cue for the deposition of building material imported from the surrounding area.

By offering the clay mixture only in the building arena outside of the nest, we were able to investigate turret construction independently of nest excavation, as workers had to actively import building material from the outside instead of just piling up the pellets excavated underground around the nest opening, a common feature of soil-nesting ants [20,27–29]. Under natural conditions, however, turrets are mainly constructed using soil pellets from underground excavation sites that are carried to the surface. It is unknown whether workers that carry soil pellets over long distances to reach the nest surface [30] may select tunnels based on their airflow direction, humidity or carbon dioxide levels. However, we recently demonstrated that leaf-cutting ants engaged in nest digging use airflow directions for orientation in the underground [31]. In such a case, the putative underground selection of environmental parameters by pellet carriers might explain why nest openings with inflowing air or no airflow hardly possess turrets in the field. Hypothetically, lack of a possibility to select tunnels with particular environmental conditions from the underground might be the reason why workers importing building material constructed a similarly-sized turret even under conditions of no airflow in our experiments.

It is important to note that we manipulated the environmental conditions only inside a single ventilation tunnel, not in the adjacent nest compartments. Consequently, changes in the building behavior of workers can only be attributed to their response to local conditions at the nest opening or inside the final tunnel section. It is an open question whether workers also react to unsuitable environmental conditions that occur inside the fungus chambers and engage in turret-building behavior. On the one side, this may appear unlikely because of the larger distances between the fungus chambers and the nest surface. On the other side, the environmental conditions inside the fungus chambers are by far steadier than those in the nest tunnels and so more reliable as cues for a long-term building response such as the construction of a turret, since airflow through the tunnels is unable to directly reach the fungus chambers [15].

### E1—Turrets constructed on nest openings containing no airflow, humid air or dry air

The presence of airflow at the nest opening hardly affected the construction of turrets, i.e., the amount of building material used by the workers was the same with or without outflowing air from the nest tunnel. In addition, the structural features of the turrets were similar irrespective of the presence of airflow, humid or dry air as turrets from all series usually only had a single large opening and a similar total turret aperture. Under natural conditions, the ants construct turrets mainly on top of nest openings located centrally on the mound. Due to the wind-induced ventilation of the nests, central openings serve as outflow tunnels, while peripheral ones serve as inflow tunnels [18]. Since turret construction usually occurs on top of openings where air leaves the nest, it is tempting to assume that workers use the airflow as a cue during the construction of ventilation turrets and deposit more pellet material around outflow channels rather than around openings where air enters the nest or no airflow is present at all. In our experiments, the amount of building material deposited by workers around the nest opening was the same for all three series and was not influenced by the presence or absence of airflow. However, turrets were higher when humid or dry air left the nest compared to those of the series without air movements, indicating that the pellets were arranged in a slightly different way around the outflow tunnel. Even though the overall building activity of the colony and the amount of material import were unaffected, workers might have preferred to drop their load in close vicinity to the perceived outflow of nest air, resulting in a deposition of pellets closer to the actual opening and thereby an increase in turret height and a decrease in turret diameter. A similar behavior was reported during nest construction in termites of the genus *Zootermopsis*, in which workers use air movements as a cue for the deposition of building material [32], and during climate control in leaf-cutting ants of the genus *Acromyrmex* [33]. In the latter, only inflow of dry air into the nest evoked the deposition of material as a building response, while inflow of humid air was not sufficient to trigger material deposition. However, it is worth noting that in both cases workers deposit material in order to seal leaks in the nest structure or close nest openings to avoid desiccation, while in *Atta vollenweideri* workers might deposit material around nest openings in order to facilitate the outflow of nest air. It is possible though that such a building response depends on other factors as well, since workers might also respond to air humidity (high vs. low), airflow direction (inflow vs. outflow) or different according to their current location (inside vs. outside the nest).

In our experiments, however, the relative humidity of the air leaving the nest tunnel had no apparent influence on turret construction, although it is known to affect the workers’ building behavior in several leaf-cutting ant species. For example, workers of *Acromyrmex heyeri* close some of the openings on their nest mound when the nest interior desiccates, i.e., when the relative air humidity drops from 98% to 50%, similar to the humidity values of 80% (humid air) and 40% (dry air) used in our experiments [34]. In *Atta vollenweideri*, humidity as well as heat loss from the nest are expected to be the reason why colonies close the majority of the nest openings during the winter months. Additionally, higher levels of air and soil moisture due to precipitation might be responsible for an increased building activity of workers on the nest surface that results in higher turrets after heavy rains [20]. In fact, the moisture of the soil has been shown to influence the excavation behavior of *Atta vollenweideri*, as workers prefer digging in moist clay with 20–22% water content, but avoid excavation in soils of lower or higher moistures [35]. Although the moisture of the clay mixture offered was constant (19% water content) in our experiments, higher air humidity might have facilitated turret construction as pellets should desiccate at a slower rate and therefore be more likely to stick to each other when workers press them into the existing turret structure. In fact, albeit not statistically significant, turret height was slightly higher when the outflowing air in the nest tunnel was humid. Partly for this reason, we used humidified air at 80% RH during the subsequent experiments.

### E2—Turrets constructed on nest openings containing different carbon dioxide levels

Size and shape, i.e., mass and height of the constructed turrets were mainly unaffected by carbon dioxide and were roughly the same for all series using carbon dioxide levels between 0.04% and 10%. Although elevated CO_2_ levels might attract ants [36] and are known to evoke digging behavior in *Solenopsis* fire ants [37], our results indicate that the release of carbon dioxide rich air from the central nest openings does not promote the construction of turrets per se. However, ants built turrets that usually contained a larger number of openings and thus a larger total aperture at higher CO_2_ levels in the outflowing air than atmospheric ones.

Leaf-cutting ants, like other underground nesting animals, are continuously exposed to elevated carbon dioxide levels of the soil air, especially in the giant underground nests of species belonging to the genus *Atta* [15,16]. An increase in carbon dioxide levels is usually accompanied by a decrease in oxygen levels. High levels of CO_2_ as those sometimes measured in field nests are known to negatively influence fungus respiration [16]; workers’ respiration is known to be affected at very low O_2_ levels [38]. Leaf-cutting ants are therefore expected to cope with such unfavorable climatic conditions in the nest by facilitating nest ventilation and thus the gas exchange with the environment.

The construction of turrets with several minor openings might be one way to improve the removal of carbon dioxide from the underground nest by increasing the turret’s open surface area. A larger opening’s total surface area should facilitate both diffusive and induced airflows and therefore increase the volume of air that can be dragged out of the nest by surface wind, thus allowing a better gas exchange with the environment as observed in ant nests with entrances of different size [13] and in the respiratory organs of animals [39]. The increase of turret aperture due to the excavation of several smaller openings was especially prominent in the series using 5% CO_2_ and to a lesser extent in the series with 1% and 10% CO_2_. A concentration of 1–2% is commonly found in well-ventilated nests of leaf-cutting ants and is therefore unlikely to be detrimental for both fungus and workers’ or brood respiration, as they are expected to have adapted to slightly increased CO_2_ levels. This could explain why the construction of several openings was not as prominent as compared to the other series containing high levels of carbon dioxide. In fact, it has been shown that workers of *Acromyrmex lundii* actually prefer slightly elevated carbon dioxide concentrations of 1–3% when relocating fungus [40].

On the other hand, a carbon dioxide concentration of 5.7% is the highest concentration that has been reported for nests of *Atta vollenweideri* [16] and it strongly indicates a poor nest ventilation, for example due to the closure of most nest openings. Although a concentration of 10% CO_2_ would indicate an even worse nest ventilation, the response of the ants was not stronger than in the 5% series. While the constructed turrets did also possess several minor openings, their total surface area was lower than in the 5% CO_2_ series. Several explanations might account for this effect. First, 10% CO_2_ exceeds by far the concentrations measured in *Atta vollenweideri* nests in the field [16] and may represent a very artificial situation. Second, while electrophysiological studies on the response characteristics of CO_2_ receptors in leaf-cutting ants revealed a working range of 0–10% CO_2_, sensory neurons showed a stimulus overload and saturation for concentrations of 7% and higher [41]. Therefore, any further increase of the carbon dioxide levels does not necessarily elicit a stronger neuronal response and might consequently cause similar behavioral reactions of workers to different stimulus intensities. Third, elevated carbon dioxide levels around 10% are known to have detrimental effects on different aspects of insect physiology [42], possibly influencing the workers’ building behavior in our experiments as well. This is further suggested by the fact that the height of the ventilation turrets tended to decrease with increasing carbon dioxide concentration. Alternatively, adding multiple openings to the ventilation turrets might simply decrease turret stability and thereby limit the potential height of the structure, leading to the construction of smaller turrets. Our results do not allow distinguishing among these possibilities and all of them might have contributed to the observed effect as well.

It is interesting to note that in our experiments, the increase in total aperture for outflowing air with 5% CO_2_ was achieved by the addition of several minor openings instead of by the enlargement of the single, initial turret opening. For similar total turret aperture, it is unlikely that a single large opening may facilitate nest ventilation stronger than several minor openings of the same total area might do, since both the overall airflow velocity and the air volume passing through the turret should be the same. However, several smaller openings might provide advantages as compared to a single, larger opening of the same surface area. The smaller the opening, i.e., the smaller the gallery across the turret wall, the easier it should be for workers to close it using soil pellets or leaf material as compared to a single large turret opening. This might allow colonies to rapidly close or re-open nest openings depending on the current ventilation needs or the temperature or humidity conditions inside the nest.

Alternatively, the addition of several small turret openings might just be a side effect of the workers’ digging behavior. When the carbon dioxide concentration inside the nest tunnel increases, workers might engage in digging behavior at the turret wall and remove previously deposited soil pellets. Depending on the diameter of the nest tunnel and the interior of the turret, one could imagine individual workers initiating excavation at separate spots, resulting in the appearance of multiple holes at different locations on the turret’s surface as opposed to the formation of a single larger opening. However, the pattern of emergence of the tunnel opening remains elusive, since we neither focused on the behavior of individual workers during turret construction, nor documented the time of the emergence of individual turret openings.

Regarding the aperture of the minor turret openings, it appeared that the size of single turret openings slightly decreased with increasing carbon dioxide concentration. It is unclear whether this effect is linked to turret stability as stated above, since increasing the carbon dioxide concentration led to an increase in the total number of openings. The unequal sample sizes between the series precluded any statistical analysis, so that the question remains unanswered so far.

In addition to the expected increase in the size of the turret opening under natural conditions, workers from field colonies might also broaden the outflow tunnels and therefore increase the volume of the nest air that can be exchanged with the environment over a given time period. Since outflow channels are not used as entrances/exits for foragers, the size of the tunnel openings should be related to climate control rather than to ant traffic. The average size of the nest openings on mounds of *Atta vollenweideri* has been reported to vary between 3 and 5 cm [20,21], yet single openings can also be quite large, with diameters up to 10 cm [23]. While in our experiments we chose the diameter of the opening and ventilation tunnel accordingly, the ants could not increase its diameter and were therefore limited to changing the opening size of the turret.

### Experiment 3 (E3)—Turrets constructed on nest openings with outflowing air containing similar carbon dioxide levels to those of the outside air

Covering the building arena, thereby removing the CO_2_ gradient at the building platform in the series using 5% CO_2_ in the outflowing air, did not affect the construction of turrets, i.e., turret height and turret mass were the same in open and closed arenas. This indicates that workers do not rely on the difference in carbon dioxide concentrations between the inside and the outside of the nest tunnel when turrets containing multiple openings are constructed.

However, the number of openings and the total aperture were different for series containing atmospheric levels compared to series containing 5% CO_2_, independent of the presence of a gradient. This confirms the results of our second experiment, where increased carbon dioxide levels caused an increase in number of turret openings as well as turret aperture.

It is known that leaf-cutting ants possess the rare ability to respond to both the absolute CO_2_ levels as well as to the changes of the carbon dioxide concentration [43] using specialized receptor cells on the tip of the antennae [44]. This feature would allow them to monitor the absolute carbon dioxide concentration in the nest continuously, indicating its biological relevance for leaf-cutting ants. In most insects, carbon dioxide serves as an orientation cue and is used for example by moths to find plants [45] or by ticks to find hosts [46]. It might be sufficient for an animal to find a certain location by following increasing CO_2_ levels compared to the background air. Although leaf-cutting ants could also use carbon dioxide to orientate in the underground, it might even be more important to assess the absolute carbon dioxide concentration instead of relying on a strong CO_2_ gradient between the inside and the outside of the nest tunnel to trigger the construction of a turret as a long-term response.

Overall, it remains unclear whether the building responses that workers showed in our experiments as a reaction to increased carbon dioxide concentrations also occur in field colonies. While turrets constructed under natural conditions may also contain several minor openings similar to the turrets built in the laboratory (Fig 1B and 1D), it is unknown whether their structural features are also related to the carbon dioxide concentration in the outflowing nest air. In addition, it needs to be investigated whether and to which extent the addition of several minor openings at high CO_2_ levels actually improves the gas exchange with the environment and enhances nest ventilation in *Atta vollenweideri* field nests.

## Supporting information

S1 File Raw data for experiments E1-E3. (XLSX)
